# Action versus Result-Oriented Schemes in a Grassland Agroecosystem: A Dynamic Modelling Approach

**DOI:** 10.1371/journal.pone.0033257

**Published:** 2012-04-05

**Authors:** Rodolphe Sabatier, Luc Doyen, Muriel Tichit

**Affiliations:** 1 INRA, UMR 1048 SADAPT, Paris, France; 2 AgroParisTech, UMR 1048 SADAPT, Paris, France; 3 CNRS, UMR 7204 CERSP, MNHN, Paris, France; University of Bern, Switzerland

## Abstract

Effects of agri-environment schemes (AES) on biodiversity remain controversial. While most AES are action-oriented, result-oriented and habitat-oriented schemes have recently been proposed as a solution to improve AES efficiency. The objective of this study was to compare action-oriented, habitat-oriented and result-oriented schemes in terms of ecological and productive performance as well as in terms of management flexibility. We developed a dynamic modelling approach based on the viable control framework to carry out a long term assessment of the three schemes in a grassland agroecosystem. The model explicitly links grazed grassland dynamics to bird population dynamics. It is applied to lapwing conservation in wet grasslands in France. We ran the model to assess the three AES scenarios. The model revealed the grazing strategies respecting ecological and productive constraints specific to each scheme. Grazing strategies were assessed by both their ecological and productive performance. The viable control approach made it possible to obtain the whole set of viable grazing strategies and therefore to quantify the management flexibility of the grassland agroecosystem. Our results showed that habitat and result-oriented scenarios led to much higher ecological performance than the action-oriented one. Differences in both ecological and productive performance between the habitat and result-oriented scenarios were limited. Flexibility of the grassland agroecosystem in the result-oriented scenario was much higher than in that of habitat-oriented scenario. Our model confirms the higher flexibility as well as the better ecological and productive performance of result-oriented schemes. A larger use of result-oriented schemes in conservation may also allow farmers to adapt their management to local conditions and to climatic variations.

## Introduction

After 15 years of implementation, the effectiveness of agri-environment schemes (AES) is still under debate [1]. Result-oriented AES have been proposed to improve the efficiency of conservation policies [2]. They rely on payment for effective biodiversity conservation (e.g. abundance, richness) independently from the management practices implemented by farmers. Such AES have been studied in the case of carnivores [3], grassland flora [4] or grassland birds [5]. If quite a large number of result-oriented schemes already exist, most of them are either experimental or have been run for too short a term and on too small a scale to be properly evaluated [2]. This situation could explain why few comparisons between result-oriented and action-oriented schemes are available and why no clear difference has been found in their effects on population sizes [6].

One of the main advantages of result-oriented schemes is to allow farmers to develop innovative management practices that would be efficient on both productive and ecological performance. By relaxing constraints on management, these schemes make it possible to implement a wider set of management strategies (i.e. sequences of management practices over time). Widening the range of management strategies may offer two advantages. First, out of the new management strategies some of them may be more efficient either on the ecological or productive performance without decreasing performance on the other dimension. Second, it may give more flexibility to the farming system [2]. Due to the difficulties of implementing and monitoring result-oriented schemes, a third kind of scheme has been created. These schemes aim at producing suitable habitat for biodiversity [7]. Their evaluation is based on indicators of habitat quality and not directly on biodiversity levels [8]. Hereafter, we will call these schemes habitat-oriented schemes. By providing suitable habitats for target species, such schemes are expected to lead to better ecological performance than action-oriented ones. However, the potential of innovation may be limited by the constraints applied on the habitat instead of on biodiversity levels. For example, result-oriented schemes allow inter-annual variability and strategies with successions of ecology-oriented and production-oriented years may appear. Moreover, these schemes may not systematically ensure good ecological performance whereas result-oriented ones should always lead, by definition, to good levels of biodiversity.

The objective of this study was to compare three scenarios corresponding to the different kinds of agri-environment schemes: action-oriented, habitat-oriented and result-oriented schemes. We first assess their differences in productive and ecological performance. A scenario will lead to better performance if it performs better in one dimension without performing worse in the other. Secondly, we explore the management flexibility linked with each scenario. A scenario will have a higher flexibility if it allows more management option than another. Finally, we illustrate the importance of management flexibility in the face of climate shock. The overall comparison of the three scenarios is based on two hypotheses:

*Hypothesis 1:* For a given result-oriented scenario, there is no habitat-oriented one that leads to better performances and for a given habitat-oriented scenario, there is no action oriented one that lead to better performances.
*Hypothesis 2:* For a given result-oriented scenario, there is no habitat oriented one that leads to a higher flexibility.

Formal definitions of these two hypotheses will be given in the core of the text.

As a case study, we focused on the conservation of lapwings *Vanellus vanellus* in wet grasslands of the French Atlantic coast (46°22′N, 1°25′W). Due to their high position in trophic networks and their close connection with wet grasslands, wader species give good information about the health of the ecosystem. The lapwing life cycle is deeply linked to the management of grassland [9] and lapwing was one of the first species to benefit from result-oriented schemes [5], [6]. Wet grasslands were the first habitats targeted by agri-environment schemes in France during the early 90's and the conservation of lapwings in these agroecosystem has long been of major concern. To compare different AES in their ability to ensure productive and ecological performance in the long term, we developed a dynamic model linking grazed grassland dynamics and lapwing population dynamics. This model focuses on the effect of AES and is thus limited to the impact of farming practices on bird dynamics. The model is built under the viable control approach [10] which is closely related to the viability theory [11]. This framework enables the satisfaction of production, socio-economic and environmental constraints and is, in this respect, a multi-criteria approach. It makes it possible to find the whole set of viable management strategies that keep a system within some constraints. As it focuses on a set of management strategies and not on a single optimal one, it is of high interest to study management flexibility, i.e. the system ability to adapt to internal or external changes. Viability analysis has been applied to biodiversity management [12], and the sustainability of agricultural systems [13], [14].

## Methods

### Model overview

**Figure 1:**
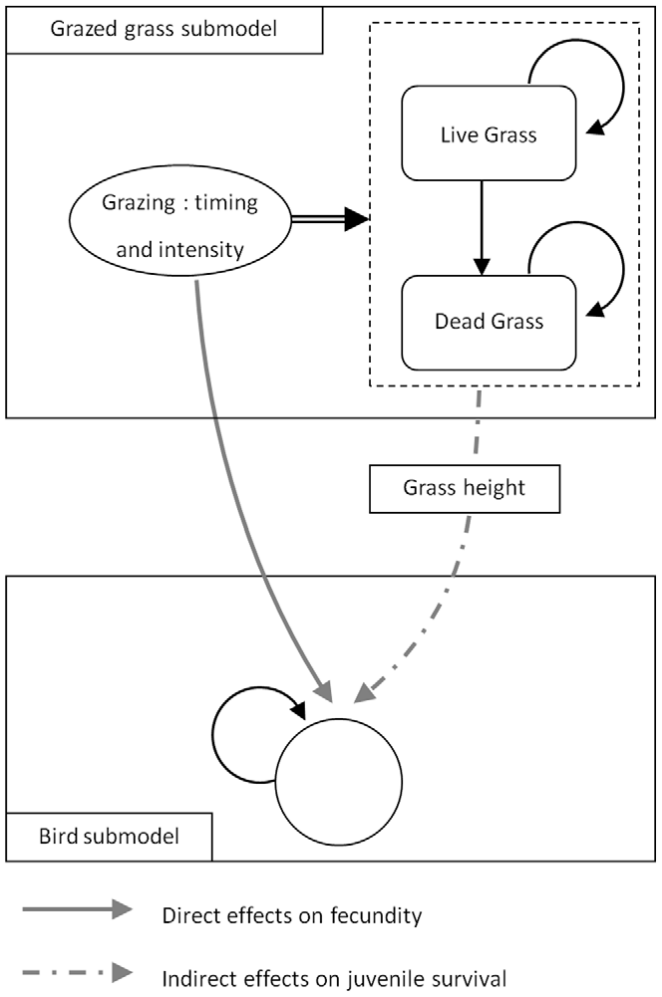
Conceptual model of the direct and indirect effects of grazing on bird population dynamics. Dynamics of grass biomass (black arrows) is controlled through timing and intensity of grazing; double arrow represents cattle consumption of standing live and dead biomass. The bird model is a single stage matrix model.

In line with the model of Sabatier, Doyen & Tichit [15], our model relies on a state-control approach that represents a grassland agroecosystem which is the breeding habitat of a bird species, the lapwing, and the feeding resource for domestic cattle. It is a discrete time model linking grazed grass dynamics to bird population dynamics (Fig. 1). Time step is defined on a monthly basis, which is coherent with farmers' management as most farmers implement middle term grazing sequences (three weeks to several months). In the grazed grass sub-model, biomass is harvested through grazing. The biomass represents a single grassland patch homogeneously managed without any spatial dimension. The grassland patch is one of the feeding resources available for cattle. We assumed that when cattle do not graze the grassland patch, they are fed elsewhere with other resources (either on temporary grasslands or indoor). The bird sub-model simulates population changes over time in response to the direct and indirect effects of grazing on bird life traits. Even if other factors than grazing may also play a role e.g. field wetness or predation, grazing indisputably remains a major factor driving the life cycle of waders (review in [9]). We therefore focus on the effects of grazing on wader dynamics. Grazing intensity has a direct effect on clutch size through nest trampling by cattle [16]. Grass height (i.e. habitat quality), generated by grazing is a key factor for foraging [17] and impacts juvenile survival. Grass height is also an important predictor of habitat nest selection [9]; however, in the absence of spatial dimension in our model, we did not model this process. The model computes two indicators summarizing the ecological and productive performance of each grazing strategy.

We studied the co-viability of the grassland agroecosystem in three scenarios (action-oriented, habitat-oriented and result-oriented scenario) by looking for viable management strategies that satisfy both ecological and productive constraints. The type of ecological constraints applied to the system differs from one scenario to the other and reflects their specificities. In the action-oriented scenario, constraints correspond to thresholds on minimal and maximal stocking density during the nesting period. Such management requirements aim at limiting the effects of trampling, while ensuring a minimum level of grazing so as to reduce grass height, heading toward better habitats. Habitat-oriented scenario combines thresholds on bird fecundity and grass heights during the chick rearing period. It ensures both fecundity and juvenile survival to be maintained to a high level. In the result-oriented scenario, constraints correspond to a threshold on minimal bird population size throughout time. Management is free and any management strategy maintaining the bird population through time is considered as viable. Productive constraints do not differ between the three scenarios. For each scenario, the model computed the viable grazing strategies meeting the constraints. The number of viable management strategies is used as an indicator of the flexibility of the system. Due to their extremely high number, viable strategies could not be counted directly and the number of states reached by the viable strategies (or size of the viability tube) was used as an index of the system flexibility.

### Grazed grass state and dynamics

The first state of the system represents a grass biomass vector *B(t)* considered monthly and partitioned into live and standing dead grass *(B_L_(t)*, *B_D_(t))* both expressed in organic matter (g OM ha^−1^). Grass dynamics is controlled by the timing and intensity of grazing *u(t)*, expressed in livestock unit per ha (LU ha^−1^). The dynamics of the grass biomass *B(t)* controlled by grazing intensity *u(t)* is summarized as follows:
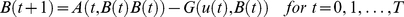
(1)where matrix *A* is a time dependent matrix that encompasses the transition rates from *t* to *t+1*. It includes grass growth, senescence and decay rates that are time dependent on a monthly basis. *G* is a vector representing biomass harvest through grazing. The state of the biomass is linked to grass height through a linear function *h(B)*. Databases from the Ouest-du-Lay marsh were used to parameterize the grazed grass dynamics ([15]; appendix S3). For further details on biomass dynamics and parameter values, see Appendix S1 and Table S1.

### Bird state and dynamics

The second state of the system describes the lapwing life cycle. By contrast with Sabatier, Doyen & Tichit [15], the bird model is deterministic and represents the female portion of a single class population. During the nesting period, cattle trampling impacts clutch size and during the chick rearing period grass height is a variation factor of juvenile survival. Assuming a pre-breeding census, the monthly dynamics of birds *N(t)* from *t* to *t+1* reads as follows:
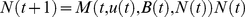
(2)where *N(t)* is the population size and *M(t,u,B,N)* the population growth function:
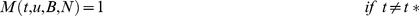
(3)

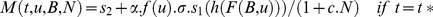
(4)with
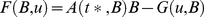
(5)where t* is the nesting month, *s_2_* the annual adult survival, *α* the proportion of breeding females, *f (u)* the clutch size depending on cattle density *u(t)*, *σ* the primary sex ratio and *s_1_ (h(F(B,u)))* the chick survival that depends on grass height *h(B)* at time t*+1. Grass height depends on grass biomass B(t*+1) and therefore on F(B(t*),u(t*)). We consider that breeding success is affected by an intra-specific competition. We use a Beverton-Holt-like density dependence function to model this competition in which *c* measures the strength of competition. A full description of the bird model along with parameter values are given in Appendix S2 and Table S2.

### Viability constraints

Three types of viability constraints formalize the multiple roles played by the grazed grassland. Constraints applied to the three scenarios are listed in Table 1.

**Table 1:** Constraint sets of the three scenarios.

		Scenarios	
Constraints	Action-oriented	Habitat-oriented	Result-oriented
Productive performance *P(u,T)>P^b^*	X	X	X
Cattle feeding requirements q.u(t)<B*(t)	X	X	X
Trampling *u(t)≤u^#^*	X		
Fecundity *f(t)≤f^b^*		X	
Grazing *u(t)≥u^b^*	X		
Habitat quality *h^b^≤h (t)≤h^#^*		X	
Population size *N(t)≥N^b^*			X

Productive performance constraint imposes that productive performance *P(u,T)* stays over a minimal productive performance *P^b^* (the minimal annual number of grazing days per hectare associated with a grazing strategy *u)*. Cattle feeding requirement constraint imposes that cattle demand *q.u(t)* is always lower than the available biomass B*(t). Cattle density constraint is an upper threshold *u^#^* on cattle density *u(t)* during the nesting month. A habitat quality constraint imposes grass height to remain within a minimal *h^b^* and maximal *h^#^* grass heights during chick rearing. Population size constraint imposes that populations size *N(t)* stays over a minimum population size *N^b^* throughout time.

#### Cattle feeding requirement constraint

Given a monthly biomass demand per livestock unit *q*, the feeding requirement constraint is defined as follows:
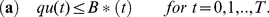
(6)


This feeding requirement constraint limits stocking density which cannot exceed the available biomass B*(t). It assumes that cattle cannot graze below a minimal biomass threshold and situations where insufficient grass availability could lead to a poorer body condition of livestock are not considered.

#### Productive constraint

A second constraint defines a minimal level of productive performance necessary for the farmer. Productive performance *P(u,T)* corresponds to the number of grazing days (simplifying to 30 days per month) associated with a grazing strategy *u = [u(0),…,u(T)]*. The model does not incorporate explicitly any spatial scale but the quantification of the productive performance is given for one hectare. The productive constraint corresponds to a lower threshold on the number of grazing days on the whole time period studied. It does not imply any minimum time period or upper threshold for grazing. It reads as follows:
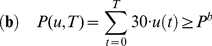
(7)where *P^b^* is the threshold of minimal productive performance. Its value was defined by the 10% lower quantile of a dataset of 344 real grazing strategies recorded on our study site [18].

#### Ecological constraints

Ecological constraints are defined in three different ways so as to capture the three kinds of scenarios.

In the action-oriented scenario (AO), the model includes two ecological constraints. The first one is related to trampling mechanisms. An upper threshold u^#^ is imposed on cattle density during the nesting month t*:
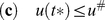
(8)


The second constraint is related to grass height during the first month following chick birth. It is represented by a lower threshold on cattle density during nesting month to induce a minimum level of grazing:
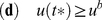
(9)


In the habitat-oriented scenario (HO), the model still includes two ecological constraints. The first one is related to clutch size in relation with trampling mechanisms. During the nesting month t*, a lower threshold, *f^#^* is imposed on clutch size *f(u)*. As *f* is a decreasing function this constraint is similar to *eqn 8*:
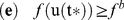
(10)


In addition to the previous constraint (eqn 10), the model also includes a constraint on habitat quality. It is imposed on grass height during the first month following chick birth (t^*^+1) in order to ensure suitable habitat for chicks. It is bounded by minimal and maximal grass heights as follows:
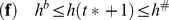
(11)


In the result-oriented scenario (RO) the model involves a single ecological constraint that imposes a minimum population size *N^b^* throughout time:
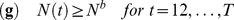
(12)


In the action-oriented scenario, the ecological constraints bound the control variable. In habitat-oriented scenario, ecological constraints combine both control and state constraints. It still limits cattle density to ensure a good clutch size and also focuses on an intermediate management objective linked with grass height to achieve a good juvenile survival. In the result-oriented scenario, no constraint is set either on cattle density or grass height and the only ecological constraint corresponds to a state constraint on the management goal which is the maintenance of the bird population size above a minimal threshold at any time step. Using such a state constraint relaxes all management restriction on farmer's decision.

### Co-viability analysis

The viability framework is used to identify combinations of biomass *B(.)*, population size *N(.)* and cattle density *u(.)* that satisfy viability constraints throughout time. It relies on the computation of the so called viability kernel [11]. In the present case, this viability kernel depends on time and we prefer to speak of a viability corridor *Viab(t).* In this section we will refer to three concepts: the viability corridor, the viable grazing strategy and the viability tube.

#### Viability corridor

The viability corridor *Viab (t)* is the set of grass biomass conditions and bird population sizes (states, *B_L_(t)*, *B_D_(t)* and *N(t)*) from which at least one grazing strategy is viable. At *t = t_0_*, the Viability corridor *Viab(t_0_)* is thus defined differently in each scenario.

In the action-oriented scenario (AO), it is defined as follows:
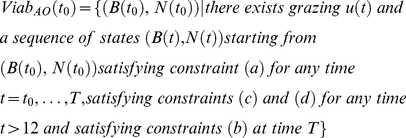
(13)


In the habitat-oriented scenario (HO), it is defined as follows:
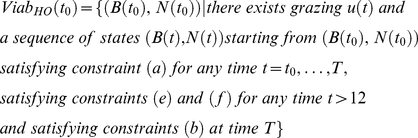
(14)


In the result-oriented scenario (RO), it is defined as follows:
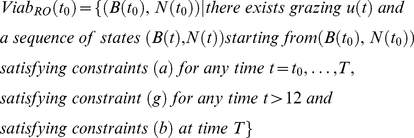
(15)


For the three scenarios, constraints (c) to (g) were not taken into account the first year (t<12) so as to enable a transition of the grazed system toward AES. This choice reflects a conventionally driven system in which AES would be introduced at the end of the first year.

#### Viable grazing strategies

Once the viability corridor has been found, we compute the viable grazing strategies that verify the different constraints over the period of time involved. Such *U* exist as long as the state *(B(t),N(t))* lies within the viability corridor *Viab(t)*. We thus consider the set of the viable grazing strategy at time *t* for a given viable state *(B(t)*, *N(t))*. A viable grazing strategy is a temporal sequence of grazing intensities that keeps the whole system within the constraint set. To each viable grazing strategy corresponds a viable state trajectory defined in terms of grass biomass and population size. These viable grazing strategies *U (t, B, N)* are defined through a dynamic programming structure.

In the action-oriented scenario (AO), it is defined as follows:
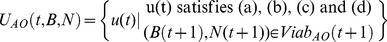
(16)


In the habitat-oriented scenario (HO), it is defined as follows:
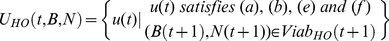
(17)


In the result-oriented scenario (RO), it is defined as follows:
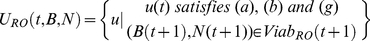
(18)


#### Viability tube

Finally, we identify the Viability tube *VT (t)*. It is the temporal succession of biomass conditions that are reachable by viable grazing strategies. It takes into account the fact that not every viable state can be reached by a viable grazing strategy. Some states are viable (i.e. starting from them, there is at least one viable grazing strategy) but they can only be reached by grazing strategies that are not viable. The viability tube is defined as follows:
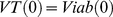
(19)

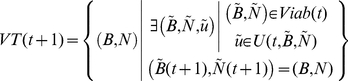
(20)As they differ among scenarios, we distinguished *VT_HO_*, *VT_RO_* and *VT_AO_*. We characterized the Viability tubes by their volumes *Θ(VT)*.
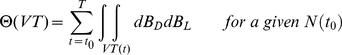
(21)
*Θ(VT)* (expressed in g^2^.s.ha^−2^) is a viability metric and an indicator of the quantity of viable state trajectories. Our system includes three state dimensions (*B_L_*, *B_D_* and *N*). So as to be able to plot the viability tubes, we limited the tubes to two states (*B_L_* and *B_D_*). The tubes therefore corresponded to projections of the 4 dimensional tubes on the three dimensional spaces defined by *B_L_*, *B_D_* and *t* for a given initial abundance *N(t_0_)*.

### Hypotheses

The two hypotheses can be formalized as follows:

*Hypothesis 1:* For a given result-oriented scenario, there is no habitat-oriented one that leads to better performances and for a given habitat-oriented scenario, there is no action oriented one that lead to better performances.
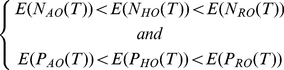
(22)whith E(N(T)) the average value of N(T) over a set of 10 000 random viable grazing strategies.
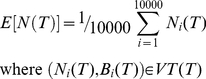
(23)Similarly, E(P(T)) is the average value of P(T) over the same set of 10 000 random viable grazing strategies.
*Hypothesis 2:* For a given result-oriented scenario, there is no habitat oriented one that leads to a higher flexibility.
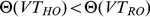
(24)The volume of the viability tube is used as an index of flexibility. A scenario leading to a bigger viability tube will allow more management strategies, and is considered being more flexible.

### Simulations

To test hypotheses 1 and 2, we followed a two step approach. First we tested them for a given set of ecological constraints and initial conditions (*u^b^ = 0.5; u^#^ = 2; f^b^ = 2.5; h^b^ = 0; h^#^ = 14; N^b^ = 30; N(t_0_) = 30*). Then we performed a sensitivity analysis to verify the generality of our results under a wider range of ecological constraints and initial conditions (*u^b^ = [0, 0.5, 1, 1.5, 2]; u^#^ = [1, 1.5, 2, 3, 4, 5]; f^b^ = [3.2, 2.5, 1.9, 1.5, 1.1]; h^b^ = [0, 5, 7, 10, 12, 13, 14]; h^#^ = [10, 12, 14, 17, 20, 30]; N(t_0_) = [25, 30, 35]*). Constraint values were chosen to explore the range of possible states and controls observed in our study area on lapwing nesting fields (0≤h≤30 and 0≤u≤5; [18]). As *f(u)* is a monotonous function of *u*, values of *f^b^* were thus chosen to correspond to the different thresholds on *u^#^*.

A dynamic programming algorithm [10] was used to identify viable initial conditions *(B(t_0_), N(t_0_))*, viable grazing strategy *U(t,B,N)*, grass state trajectories *B(t)* and bird population state trajectories *N(t)* respecting the different constraints at each time step over a period of *T* = 96 months. The numerical computations were performed with Scilab 4.1.2 software (http://www.scilab.org/; Scilab Consortium 2007). Once viable grazing strategies and state trajectories were found, their ecological and productive performances *N(T)* and *P(T)* were assessed. The performance of the agroecosystem under the three scenarios AO, HO and RO was compared with a permutation tests using Python 2.6 (http://www.python.org/) so as to test Hypothesis 1. For a given performance (ecological or productive one) and for a given pair of scenarios, the test calculates a criterion (the difference of the average performances) and compares it to the distribution of this criterion for n = 10000 random permutations within the two sets of trajectories tested. The p value of the test is the proportion of permuted situations for which the criterion is larger (in absolute value) than the criterion of the not permuted situation. More details on permutation tests can, for example, be found in [19]. In order to investigate the advantage of the improved flexibility of the result-oriented scenario in facing climatic variations, we tested the effect on the viability tubes of a shock in climatic conditions represented by an increased grass growth in year 5. Parameters of matrix A were modified so as to simulate an earlier grass onset in the season (i.e. one month earlier) and a stronger grass growth (i.e. +25%).

## Results

### Hypothesis 1: scenarios differ in performance

**Figure 2:**
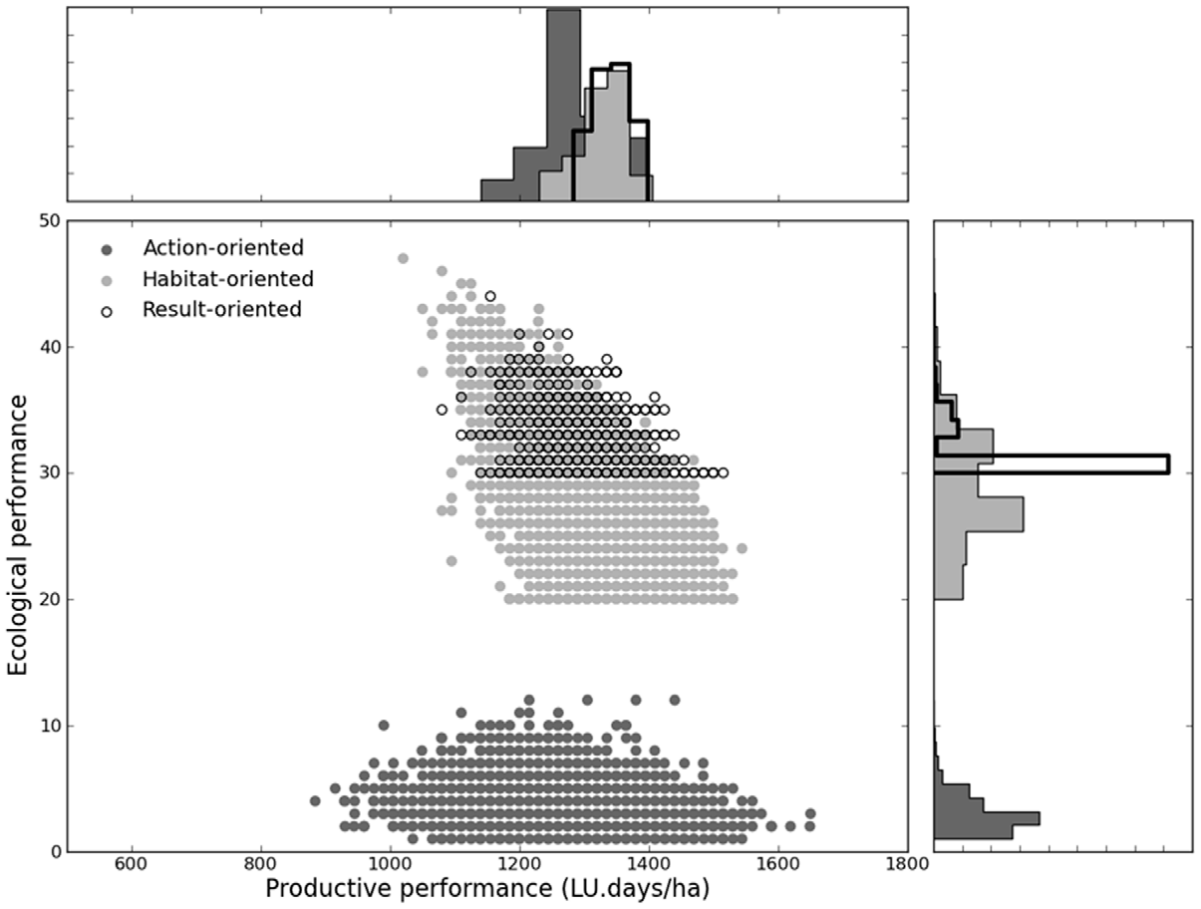
Ecological performance *N(T)* and productive performance *P(u,T)* and histograms of distributions of action-oriented, habitat-oriented and result-oriented. For each scenario, results are plotted for a sample of 10 000 couples of viable state trajectories and viable grazing strategies. The action-oriented scenario (dark gray) is run with cattle density constraint (*u^b^* = 0.5; *u^#^* = 2 livestock units per hectare); the habitat-oriented scenario (light gray) is run with fecundity and habitat quality constraints (*f^b^* = 2.5, *h^b^* = 0 cm and *h^#^* = 14 cm); the result-oriented scenario (empty black) is run with minimum population size (*N^b^* = 30); all, scenario involve constraints on productive performance and cattle feeding requirement; all scenarii are run with initial population size *N(t_0_)* = 30.

Fig. 2 shows the ecological and productive performance of a sample of 10 000 grazing strategies for each of the three scenarios. Comparison of both average ecological and productive performance of the three scenarios showed significant differences (permutation test, p-value = 0). However, differences between the habitat and result-oriented scenarios were much lower than differences between the action-oriented scenario and the other two scenarios (Table 2). The result-oriented scenario led to better performances than the habitat-oriented one and the latter scenario led to much better ecological performance than the action-oriented one and slightly better productive ones. However, it should be kept in mind that the habitat and result-oriented scenarios were very similar for both performance criteria.

**Table 2:** Ecological and productive performance of action, habitat and result-oriented scenarios.

	Action oriented	Habitat oriented	Result oriented
Productive performance(LU.days/ha)	1313(95)	1321(74)	1339(58)
Ecological performance(Population size)	4(1)	29(4)	31(2)

Means and standard deviation () are given for three random samples of 10 000 viable state trajectories and viable grazing strategies. Productive performance *P(u,T)* is the number of livestock unit.days ha^−1^ (LU.days/ha) characterizing a grazing strategy. The ecological performance *N(T)* is the bird population size at time horizon (starting with N(t_0_) = 30).

### Hypothesis 2: the result-oriented scenario improves management flexibility

We restricted the comparison of flexibility to the other two scenarios since the action-oriented scenario did not maintain bird populations. The inclusion of the tubes, their shape and their volumes showed that more states and controls were viable in the result-oriented scenario than in the habitat-oriented one. Numerical computations showed that the habitat-oriented tube was included in the result-oriented one:
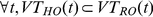
(25)


**Figure 3:**
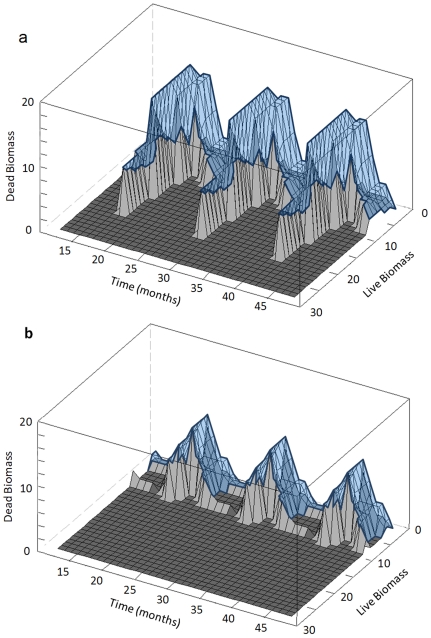
Zoom on three years of the viability tubes (*VT_RO_* and *VT_HO_*) for the result-oriented (fig a) and habitat-oriented (fig b) scenarios. The tubes show the set of viable states throughout time (in months). The two state dimensions are the live biomass and the dead biomass both expressed in organic matter (10^−4^ g OM ha^−1^). The viability tube corresponds to the volume (in blue) between the light gray surface and the wireframe. Dark gray areas are the ones for which no viable state exists.

The inclusion of the two tubes means that the flexibility of the result-oriented scenario at least as high as the flexibility of the habitat-oriented one. For these two scenarios, ensuring similar levels of performance (Table 2), tubes were bigger in the result-oriented than in the habitat-oriented scenario. Indeed, the calculation of *Θ(VT)* showed 1.5 more viable grass states in the result-oriented scenario than in the habitat-oriented one (*Θ(VT_RO_)* = 6842 versus *Θ(VT_HO_)* = 4997 g^2^.s.ha^−2^). A larger range of grass biomass conditions was thus available for farmers throughout time. The shape of the *Viability tube* for both habitat and result-oriented scenarios illustrates the couples of possible viable states *(B_L_, B_D_)* throughout time and the higher flexibility of the result-oriented scheme (Fig. 3.a and 3.b).

These results illustrate that more flexibility was given to the grazing strategies in the result-oriented scenario. We have therefore validated Hypothesis 2. In terms of management this means that the farmer could implement a wider range of grazing strategies in the result-oriented scenario than in the habitat-oriented one (appendix S4). Especially, higher cattle densities can be implemented in spring with the result-oriented scheme.

### Sensitivity analysis

Results of the sensitivity analysis are presented in Appendix S5. Sensitivity analysis showed one limit case (h^#^ = 30 cm) for which Hypothesis 1 was falsified. In this situation both performances of the action-oriented scenario were higher than those of the habitat-oriented one. Apart from this case, when scenarios could be ranked, action-oriented scenario always led to worse performances than habitat-oriented one and both action and habitat-oriented scenarios led to worse performances than result-oriented scenario. Hypothesis 1 was therefore acceptable for most constraint values. Whatever the parameter settings, Hypothesis 2 was always true.

### Illustrating the importance of flexibility

**Figure 4:**
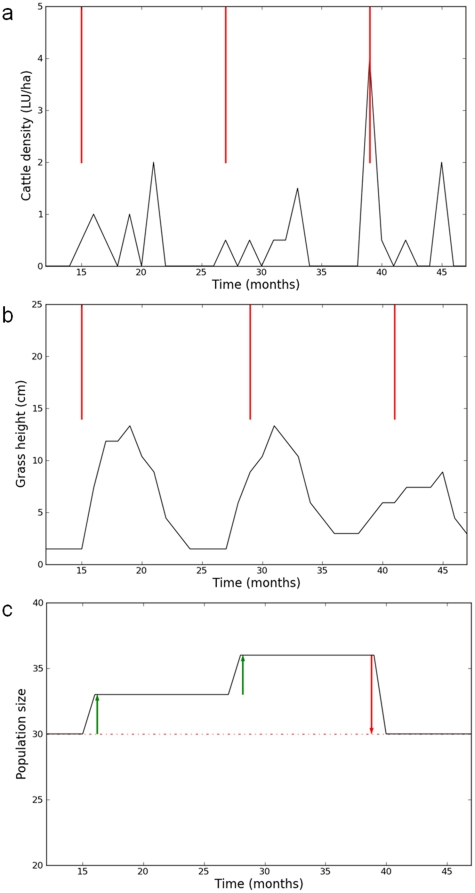
Example of one viable grazing strategies and state trajectories in the result-oriented scenario with a climatic perturbation (zoom on the three years around the climatic perturbation). The different constraints are plotted to illustrate the fact that viable result-oriented strategies would not respect action and habitat oriented constraints. Figure a, viable grazing strategy in the result oriented scheme. Red bars represent the cattle density constraint *u^#^* applied in the habitat and action oriented schemes. Figure b, viable grass height trajectory *h* in the action and result oriented schemes. Red bars represent the grass height constraint *h^#^* applied in the habitat and action oriented schemes. Figure c, viable bird population trajectory *N*. The red dotted line stands for the population size constraint *N^b^*. The green and red arrows highlight the mechanisms of inter-annual compensation.

We examined the interest of the improved flexibility of the result-oriented scenario in facing environmental variations. It turned out that the state of the system still lied within the result-oriented viability tube *VT_RO_* despite the disturbance, while it left the habitat-oriented viability tube *VT_HO_*. In other words, no couple of control strategy and state trajectory respected all productive and habitat-oriented ecological constraints. Thus it was not possible for the farmer to produce a suitable grass height for birds every year with low trampling while ensuring good productive performance and satisfying cattle feeding requirements. However, the result-oriented tube was not empty and it was possible to find viable state trajectories and control strategies. As illustrated with one simulation (Fig. 4), a viable result-oriented grazing strategy did not respect habitat-oriented constraints every year but it did, however, maintain bird populations throughout time due to inter-annual compensations. In this example, grazing intensity in spring was low in 2009 and 2010 (Fig. 4.a). It implied low levels of trampling and an increase in bird population sizes (Fig. 4.c). In 2011, spring grazing intensity was stronger and bird population decreased but still remained above the population threshold. This result shows how, in the result-oriented scenario, the farmer can adapt his management to climatic shocks by implementing an inter-annual variation of management strategies. Such inter-annual variation in management was not available in the habitat-oriented scenario. This result again emphasized the advantages of the increased flexibility provided by the result-oriented scenario.

## Discussion

First, our results showed that in most cases the habitat and result-oriented scenarios led to much better ecological performance than the action-oriented scenario. Productive performance was quite similar among the scenarios. Secondly, our results showed that the result-oriented scenario had a higher flexibility than the habitat-oriented one. This difference in flexibility was even greater when the grazed grassland agroecosystem was exposed to climatic variation.

### A modelling approach to compare management schemes

Using a modelling approach gave us the opportunity to compare situations all other things being equal, as we would have done in a controlled trial. We therefore did not include mechanisms such as environmental stochasticity or landscape source/sink mechanisms. These mechanisms are of high importance in the real world but management through grazing has low (if any) impact on them and including them in the model would only have blurred the simulation results. These considerations have to be kept in mind when considering the results. As an example, one of the main differences between the model and reality is the absence of migration. Here, we considered a closed population of birds to assess the effects of management practices. Using population size as an indicator of ecological performances was therefore possible as well as very convenient and illustrative. In the field, such an indicator would raise questions. In the one hand it does not only reflect mechanisms occurring at field scale but in the other hand, this indicator is much closer to the final objective of a conservation policy than a bird productivity index would be.

Our results showed several undetermined situations. They could occur for two reasons: either the three scenarios could not be ranked or it was impossible to find any viable grazing strategies. Changing the values of the constraints oriented the set of viable strategies to either better ecological or better productive performance, illustrating the trade-off between production and conservation in such agroecosystems [15], [20]. It could lead to extreme situations with very high performances on one dimension and very low on the other dimension. These situations could not be put in a hierarchy. In other cases, the constraint values tested pushed the system too far and no viable grazing strategy could be found. Consequently, nothing could be said on Hypothesis 1 since no performance could be assessed.

Result-oriented schemes aim at protecting the whole agroecosystem by targeting umbrella species. We could here focus on management strategies that impact the whole agroecosystem and offer advantages to other species with similar ecological requirements and similar sensitivity to management. However, in the field, farmers may implement very specific measures only benefiting the target species. For example, in the result-oriented scheme implemented in the Netherlands, it happened that farmers only build an electric fence around the nest [5]. If this management leads to better hatching success for the target species, it is of minor interest for other species in the agroecosystem. This measure has been strongly criticized for its lack of cost effectiveness [21] and was cancelled in the new scheme. To avoid it, the evaluation of management must be done on an indicator as close to the final objective of conservation as possible. Considering several species [8], [22] could be a powerful solution. Best effects are expected with management options having broad effects on the agroecosystem. In this respect, management options at field scale include grazing sequences, amount and timing of fertilization as well as mowing techniques and dates. At upper scale, the proportion of land uses [23], [24] as well as their spatial arrangement [25], [26], [27] could also be efficient management options that would impact the whole agroecosystem.

### Improving management flexibility

Multi-criterion analysis mainly looks for optimal performance but do not take into account the issue of flexibility in decision making. Optimality is well adapted to static situations or stable environments but flexibility is of major concern for systems exposed to uncertainties [28]. The viable control approach makes it possible to go beyond the search of optimum and to look for a diversity of management strategies. Although management strategies were quite similar in terms of performance, the number of viable management strategies gave a strong advantage to result-oriented schemes. Greater flexibility of management is one of the major arguments in favour of result-oriented schemes [2]. First, it is expected to improve the resilience of the agroecosystem as farmers may choose alternative management strategies to adapt to inter-annual climatic variability. The agroecosystems we studied are low input, extensively grazed grasslands. Such systems are highly dependent on climatic conditions and flexibility in grassland use is a major component in coping with unexpected events [29]. In comparison with habitat-oriented schemes that impose constraints on habitat and fecundity every year and forces periodic management strategies, the result-oriented schemes allow for inter-annual variability. It gives the possibility of segregating ecological and productive objectives among years (e.g. to adapt grazing strategies to climatic conditions). These new strategies are the basis of the higher flexibility of the result oriented schemes. Our study focussed on temporal flexibility of grazing strategies but we conjecture that in the same way, spatial flexibility would allow farmers to adapt their management to variations in external conditions among several fields. Further development of the model will take these spatial variations into account. A second advantage of this greater flexibility would be to allow farmers to look for innovative management strategies. Our results suggest that loosening the ecological constraints of the agroecosystem gives farmers a higher degree of freedom. Matzdorf & Lorenz [30] indicate that this potential of innovation is very well used by farmers involved in result-oriented schemes. It also leads the farmers to become more involved in conservation and increases their willingness to improve ecological performance of their fields [2]. In this study, we focussed on a well known species. However, such detailed knowledge is not often available. In the absence of stabilized knowledge on the effects of farming activities on biodiversity, the high potential of innovation, associated to the willingness to improve ecological performance that result-oriented schemes provide may help finding ecological sound management strategies. In such a context, biodiversity becomes a joined-production that could be considered as a new “crop” and the capacity of farmers (in link with local environmental managers and/or researchers) to produce the empirical knowledge needed should not be underestimated. In this transition phase, the modalities of the compensation payments may however be reconsidered and a form of payment for knowledge production could replace the payment for results. In the model, such an imperfect knowledge could be integrated by adding uncertainty on the key parameters in the form of stochasticity. Using algorithms of stochastic viability [10] would make it possible to maintain the viability approach in such a context.

Result-oriented schemes have many advantages. They seem moreover to be very well accepted by farmers since they do not necessarily imply extra-costs and allow for more room for manoeuvre in the management of their farm [8]. The set up of such schemes in the field seems to be more limited by legal issue than by acceptance by local stakeholders. Indeed, the Rural Development Regulation, based on a strict interpretation of the World Trade Organisation rules, restricts payments for farmers to compensations of income losses or additional costs due to a change of management practice. This rule fits well to Action-Oriented Schemes but result-oriented ones are seen as distorting measures and public stakeholders are often reluctant to implement them. This legal problem is one of the reasons for the abandonment of the Dutch result-oriented scheme [31]. According to Schwartz et al. [2], a window of negotiation seems however to be available in the WTO rules but would imply high level negotiations.

### Toward increased spatial scales

Other mechanisms may improve the effectiveness of result-oriented schemes. For instance, farmers frequently allocate schemes to fields with the lowest productivity so as to limit the impact on the overall performance of the farm [2]. Therefore, the localisation of AES fields is often defined regardless of its expected ecological outcome. With result-oriented schemes both productive and ecological performance would have to be taken into account as the ecological outcome would be of major concern to farmers. Such schemes could thus be expected to reach better levels of effectiveness. The level of payment would however need to be addressed with caution for the scheme to remain attractive. Our model does not include economic incentives yet and development in this direction should help defining these levels of payment.

Beyond the legal issues mentioned at the end of the former section, other limits of result-oriented schemes arise from the possible difficulties to assess the ecological outputs. Schwarz *et al.* [2] recommend focusing in a first step on plant communities as ecological and agricultural processes fit into the same scale: the field. Methods that prove to be fair to the farmer have been developed in Germany [22] and in France [8] to provide assessments in the case of grassland flora. However, concerning mobile species, such as birds, with larger home ranges, assessment at field scale is more difficult. First, birds are not present in the field all the time and accurate surveys imply heavy monitoring protocols. A solution to this first problem was to focus on local indicators such as breeding success but results were mitigated. [5], [6]. The second difficulty, which is linked to the latter point, is that bird population trends not only depend on processes occurring at the field scale but also on processes occurring at a larger scale (i.e. a set of neighbouring fields). A solution to this problem could be to develop schemes at a scale matching the home range of species under concern. However, management at larger scales involving several land owners may lead to situations where some land owners behave as free-riders and compromise the success of the scheme. This issue has been taken into consideration in Sweden in the case of carnivores with very large home ranges [3]. In this case, payments by results were not given directly to individuals but to the communities. The efficiency of the conservation policy thus relied on collective action. Result-oriented schemes at the landscape scale based on collective action would have another major advantage. Groups of farmers could both adapt their management practices at the field scale and modify the spatial allocation of management practices at the landscape scale in order to create habitat heterogeneity. Increased landscape heterogeneity could improve ecological performance as it makes spatial complementarities among habitats possible [26]. Improvement of the model presented here to account for these spatial effects (nest site selection, landscape heterogeneity,…) is another major perspective of this work that we are currently handling [27], [32].

### Conclusion

Compared with action-oriented schemes, our study shows that improvement of ecological performance is high when schemes are habitat or result-oriented. Differences in performances between habitat and result-oriented schemes remained limited. The main advantage of result-oriented schemes is to increase the overall management flexibility of the grassland agroecosystem. Such improved flexibility may also allow farmers to adapt their management to climatic variations. Further model developments will focus on both the spatial and temporal dimensions of farming flexibility. This next step will make it possible to better match management and ecological processes.

## Supporting Information

Appendix S1 **Discrete time dynamics of the grazed grassland.**
(DOC)

Appendix S2 **Discrete time dynamics of the wader population.**
(DOC)

Appendix S3 **Model calibration.**
(DOC)

Appendix S4 **Different degrees of freedom in grazing sequences.**
(DOC)

Appendix S5 **Sensitivity analysis.**
(DOC)

Table S1 **Parameters used in the grazed grass model.**
(DOC)

Table S2 **Parameters used in the bird model.**
(DOC)
