# Supplementary material for: Action versus Result-Oriented Schemes in a Grassland Agroecosystem: A Dynamic Modelling Approach
Source: PLoS One. 2012 Apr 5;7(4):e33257. doi: 10.1371/journal.pone.0033257 (PMC3320605; doi:10.1371/journal.pone.0033257)
Supplement: Appendix S1 — Discrete time dynamics of the grazed grassland. (DOC) [file pone.0033257.s001.doc]

**Appendix S1.** Discrete time dynamics of the grazed grassland

The grass biomass *B(t)* is partitioned into live and standing dead grass *(B_L_(t), B_D_(t))* both expressed in organic matter (g OM ha^-1^). Grazed grass dynamics read:

*B(t+1)=A(t,B(t))=A(t,B(t))-G(u(t),B(t))* *for t = 0,1,…,T* (eqn S1)

where matrix *A* encompasses the transition rates defining grass dynamics and *G* is a vector representing the biomass harvested through grazing. Matrix *A* is specified as follows:

$A(t,B)=\left(\begin{matrix} \exp(-r_{S}(t))+r_{G}(B,t) & 0 \\ 1-\exp(-r_{S}(t)) & \exp(-r_{D}(t)) \end{matrix}\right)$ (eqn S2)

- *r_S_(t)* and *r_D_(t)* (month^-1^) stand for the senescence and decay rate coefficients.

- growth rate *r_G_(B, t)* (g OM month^−1^) is the product of a potential growth rate *γ_1_(t)* (g m^−2^ month^−1^), also time dependent, and the relative light interception by live mass based on Beer’s law:

$r_{G}\left(B,t\right)=\frac{\gamma_{1}\left(t\right)}{B_{L}+B_{D}}\left(1-\exp\left(-\beta\mu\left(B_{L}+B_{D}\right)\right)\right)$ (eqn S3)

where γ_1_(t) is the maximum possible per month increase in grass mass under ideal environmental conditions (i.e. without nitrogen or water limitation), β an attenuation coefficient related to sun angle and μ is a specific leaf area (m² gOM^−1^).

Harvest through grazing represents the control variable. It depends on grazing intensity *u(t)*, expressed in Livestock Unit per square meter. Each *LU* requires a certain amount *q* of grass per month corresponding to the unit feed requirement in terms of biomass of one livestock unit on a monthly basis (gOM month^-1^). We assume that biomass harvest through grazing operates with a preference for live grass:

$G\left(u(t),t\right)=\left\{\begin{matrix} G_{L}\left(t\right)=\min\left(qu,B*_{L}\left(t\right)\right) \\ G_{D}\left(t\right)=qu\left(t\right)-G_{L}\left(t\right) \end{matrix}\right.$ (eqn S4)

with *B*(t)=A(t,B(t)) B(t)*

The state of grass biomass determines grass height which is denoted *h(B)*.
