# Supplementary material for: Action versus Result-Oriented Schemes in a Grassland Agroecosystem: A Dynamic Modelling Approach
Source: PLoS One. 2012 Apr 5;7(4):e33257. doi: 10.1371/journal.pone.0033257 (PMC3320605; doi:10.1371/journal.pone.0033257)
Supplement: Appendix S2 — Discrete time dynamics of the wader population. (DOC) [file pone.0033257.s002.doc]

**Appendix S2.** Discrete time dynamics of the lapwing population

The lapwing life cycle, impacted by the direct and indirect effects of grazing, is described. During the nesting period, cattle trampling impacts clutch size and during the chick rearing period grass height is a variation factor of juvenile survival. Assuming a pre-breeding census, the monthly dynamics of birds *N(t)* from *t* to *t+1* reads as follows:

*N(t+1) = M(t,u(t),B(t),N(t)) N(t) (eqn S5)*

where *N(t)* is the population size and *M(t,u,B,N)* the population growth function

*M (t,u,B,N)=1 if t≠t* (eqn S6)*

*M (t,u,B,N)= s_2 +_  α.f (u) . σ . s_1_(h(F(B,u))) / (1 + c . N) if t=t* (eqn S7)*

with $\text{F(B,u)}=A(t*,B)B-G(u,B)$

where *t** is the nesting month, *s_2_* the adult survival, *α* the proportion of breeding females, *f (u)* the clutch size depending on cattle density *u(t)*, *σ* the primary sex ratio and *s_1_(h(F(B,u)))* the chick survival that depends on grass height *h(B)* at time *t*+1*. Grass height depends on grass biomass *B(t*+1)* and therefore on *F(B(t*),u(t*))*.

We consider that breeding success is affected by intra-specific competition. We use a Beverton-Holt-like density dependence function to model this competition in which *c* measures the strength of competition.

Clutch size is influenced by grazing intensity through nest trampling by cattle. With *η* the effect of one livestock unit on the daily survival rate of eggs, the egg survival rate over the whole incubation period *x* is assumed to depend on cattle density *u* and incubation length *t^inc^* as follows:

$x(u,t^{inc})=\eta^{u.t^{inc}}$ *(eqn S8)*

Therefore, clutch size reads:

$f(u)=x(u).f^{\max}=\eta^{u.t^{inc}}.f^{\max}$ *(eqn S9)*

with *f ^max^* being the maximal fecundity without trampling.
