# Supplementary material for: Action versus Result-Oriented Schemes in a Grassland Agroecosystem: A Dynamic Modelling Approach
Source: PLoS One. 2012 Apr 5;7(4):e33257. doi: 10.1371/journal.pone.0033257 (PMC3320605; doi:10.1371/journal.pone.0033257)
Supplement: Appendix S3 — Model calibration. (DOC) [file pone.0033257.s003.doc]

**Appendix S3.** Model calibration

*Grazed grass sub-model*

Three databases from the Ouest-du-Lay marsh were used to parameterize the grazed grass dynamics. A harvesting trial was conducted on 15 grazed fields to derive the relationship between grass height and biomass. In 2004, three measurements were taken (February, April, May). In each field, grass height and biomass were measured on 16 randomly positioned quadrats (0.0625m²) and then averaged. In each quadrat 9 grass height measurements (to the nearest cm) were taken using a sward stick. Measurements were distributed every 8 cm, with the initial point being located at 4.5 cm from the edges of the quadrat. The above-ground grass inside each quadrat was harvested using a hand-mower, dried at 80°C for 24 h and weighed to the nearest gram. For each field, the three measurements were averaged. The relationship between grass height (*h*) and grass biomass (*B*) was modelled with a linear regression with no intercept (*lm* procedure R software) (Table 1).

A second database was used to calibrate grass growth (*r_G_*), scenescence (*r_S_*) and decay (*r_D_*) rate vectors without grazing. We used three trials of grass height measurements on 74 ungrazed fields with a continuous development of grass in spring (Durant et al., 2008a). In each field grass height measurements (20 sward stick measurements ha^-1^ to the nearest cm) were taken in February, April and May, providing respectively the initial state of the grass cover, the beginning of the growing period and the maximal growth. The calibration was done by minimizing the mean square error (MSE) between predicted and measured grass heights (Table 1). A third database (Tichit et al., 2005a) was used to calibrate the cattle unit feed requirement *q*. It included the sequences in grazing intensities and the grass heights (10 measurements with the above mentioned method) recorded on 12 fields in 2002. Calibration was also performed by minimizing the MSE. A final MSE of 27 cm² was obtained which correspond to a 5.2 cm root mean squared error.

*Bird sub-model*

Wader demographic parameters were based on literature data. Most reviews of wader demography emphasize low reproductive success (mean clutch size around 4 eggs, high nest failure, 1 brood per year), and relatively high adult survival varying from 0.7 to 0.9 (Sandercock, 2003). We used average demographic parameters of lapwings and redshanks which are representative of these general characteristics (Table 2). Competition between chicks was assumed to be intraspecific and parameters *c_i_*, measuring the strength of competition, was set at 0.0077 for both species (Tichit et al., 2007). Due to the lack of precise data about the relationship between chick survival and grass height, a threshold approach was used (Tichit et al., 2007; Fig S3.1). Mean chick survival given in the literature was used for the viable grass height range (0-14cm). It was divided by four out of this range while grass height remained close to the viable grass height range (14-20cm) and set to almost zero (0.001) further from the viable grass height range (>20cm).

**S_0_(h)**

**Grass height (h)**

Fig S3.1: Threshold function used to asses the effect of grass height on juvenile survival.
