# Supplementary material for: Action versus Result-Oriented Schemes in a Grassland Agroecosystem: A Dynamic Modelling Approach
Source: PLoS One. 2012 Apr 5;7(4):e33257. doi: 10.1371/journal.pone.0033257 (PMC3320605; doi:10.1371/journal.pone.0033257)
Supplement: Appendix S4 — Different degrees of freedom in grazing sequences. (DOC) [file pone.0033257.s004.doc]

**Appendix S4.** Different degrees of freedom in grazing sequences

Grazing intensity (LU/ha)

**a.**

Time (year)

Grazing intensity (LU/ha)

**b.**

Time (year)

Set of 10 random viable trajectories in the habitat-oriented scenario (a) and the result-oriented scenario (b).
