# Supplementary material for: Action versus Result-Oriented Schemes in a Grassland Agroecosystem: A Dynamic Modelling Approach
Source: PLoS One. 2012 Apr 5;7(4):e33257. doi: 10.1371/journal.pone.0033257 (PMC3320605; doi:10.1371/journal.pone.0033257)
Supplement: Appendix S5 — Sensitivity analysis. (DOC) [file pone.0033257.s005.doc]

**Appendix S5: Sensitivity analysis**

*Hypothesis one: performance*

Table S5.1 shows the average ecological and productive performance of samples of 10 000 grazing strategies for each of the three scenarios for different parameter settings of ecological constraints and initial conditions. Not all scenarios included viable grazing strategies. Logically, the result-oriented scenario maintained population sizes in all cases. When viable grazing strategies existed, the habitat-oriented and action-oriented scenarios did not maintain population size for every grazing strategy. Comparison of performance always showed significant differences (T-test, P-value<10^-15^ for each pair of scenario compared). Even though we observed several cases for which it was not possible to put scenarios into a hierarchy, action or habitat-oriented scenarios never led to better performance than the result-oriented scenario on both dimensions. In most cases, the action-oriented scenario led to lower performances than the habitat-oriented one. However when constraints on habitat were strongly relaxed (h^#^=30cm), habitat-oriented scenario led to lower performances than the action-oriented one. On the management point of view, this constraint can be considered as very weak because 10% of grazed fields only had higher grass heights at this time of the year (Durant et al. 2008b).

Sensitivity to N(t_0_) showed a similar situation (Table S5.1.). Hypothesis 1 remained true with an increased value of N(t_0_). However with a lower value of N(t_0_), it was not possible to rank the three scenarios as the habitat-oriented scenario performed better than the result-oriented one on the productive dimension but worse on the ecological one.

*Hypothesis two: flexibility*

The size of the viability tubes could vary with the different parameter values but result-oriented scenario always showed a much larger viability tube than habitat-oriented ones thus confirming Hypothesis 2 (Table S5.2).

**Table S5.1.** Model sensitivity to different settings of parameter values. Average ecological and productive performance for action-oriented (AO), habitat-oriented (HO) and result-oriented (RO) scenarios. For each set of parameters, hypothesis 1 was either qualified as True, False or Undetermined. Results corresponding to the set of parameter used in the first step of the study are shown in *italic*.

a. sensitivity to u^b^

|  | Productive performance | | | Comparison | | | Ecological performance | | | Comparison | | | Hypothesis 1 |
| --- | --- | --- | --- | --- | --- | --- | --- | --- | --- | --- | --- | --- | --- |
| u^b^ | AO | HO | RO | AO<HO | HO<RO | AO<RO | AO | HO | RO | AO<HO | HO<RO | AO<RO |
| **0** | 1277 | 1320 | 1339 | True | True | True | 3.4 | 28.4 | 31 | True | True | True | **TRUE** |
| ***0.5*** | *1313* | *1320* | *1339* | *True* | *True* | *True* | *3.6* | *28.4* | *31* | *True* | *True* | *True* | ***TRUE*** |
| **1** | 1360 | 1320 | 1339 | False | True | False | 4.0 | 28.4 | 31 | True | True | True | **Undetermined** |

b. sensitivity to u^#^

|  |  | Productive performance | | | Comparison | | | Ecological performance | | | Comparison | | | Hypothesis 1 |
| --- | --- | --- | --- | --- | --- | --- | --- | --- | --- | --- | --- | --- | --- | --- |
| u^#^ | f^#^ | AO | HO | RO | AO<HO | HO<RO | AO<RO | AO | HO | RO | AO<HO | HO<RO | AO<RO |
| **1** | **3.2** | 1230 | - | 1339 | NA | NA | True | 2.2 | - | 31 | NA | NA | True | **Undetermined** |
| **1.5** | **2.9** | 1230 | *-* | 1339 | NA | NA | True | 2.2 | *-* | 31 | NA | NA | True | **Undetermined** |
| ***2*** | ***2.5*** | *1313* | *1320* | *1339* | *True* | *True* | *True* | *3.6* | *28.4* | *31* | *True* | *True* | *True* | ***TRUE*** |
| **3** | **1.9** | 1348 | 1334 | 1339 | False | True | False | 3.8 | 26.2 | 31 | True | True | True | **Undetermined** |
| **4** | **1.5** | 1443 | 1632 | 1339 | True | False | False | 4.9 | 11.4 | 31 | True | True | True | **Undetermined** |
| **5** | **1.1** | 1495 | 1679 | 1339 | True | False | False | 5.3 | 9.5 | 31 | True | True | True | **Undetermined** |

c. sensitivity to h^b^

|  |  | Productive performance | | | Comparison | | | Ecological performance | | | Comparison | | | Hypothesis 1 |
| --- | --- | --- | --- | --- | --- | --- | --- | --- | --- | --- | --- | --- | --- | --- |
| h^b^ | h^#^ | AO | HO | RO | AO<HO | HO<RO | AO<RO | AO | HO | RO | AO<HO | HO<RO | AO<RO |
| ***0*** | ***14*** | *1313* | *1320* | *1339* | *True* | *True* | *True* | *3.6* | *28.4* | *31.0* | *True* | *True* | *True* | ***TRUE*** |
| **5** | **14** | 1313 | 1319 | 1339 | True | True | True | 3.6 | 28.5 | 31.0 | True | True | True | **TRUE** |
| **7** | **14** | 1313 | 1324 | 1339 | True | True | True | 3.6 | 28.3 | 31.0 | True | True | True | **TRUE** |
| **10** | **14** | 1313 | 1386 | 1339 | True | False | True | 3.6 | 25.2 | 31.0 | True | True | True | **Undetermined** |
| **12** | **14** | 1313 | 1366 | 1339 | True | False | True | 3.6 | 28.7 | 31.0 | True | True | True | **Undetermined** |
| **13** | **14** | 1313 | 1367 | 1339 | True | False | True | 3.6 | 28.7 | 31.0 | True | True | True | **Undetermined** |

d. sensitivity to h^#^

|  |  | Productive performance | | | Comparison | | | Ecological performance | | | Comparison | | | Hypothesis 1 |
| --- | --- | --- | --- | --- | --- | --- | --- | --- | --- | --- | --- | --- | --- | --- |
| h^b^ | h^#^ | AO | HO | RO | AO<HO | HO<RO | AO<RO | AO | HO | RO | AO<HO | HO<RO | AO<RO |
| **0** | **10** | 1313 | - | 1339 | - | - | True | 3.6 | - | 31.0 | - | - | True | **Undetermined** |
| **0** | **12** | 1313 | 1335 | 1339 | True | True | True | 3.6 | 26.2 | 31.0 | True | True | True | **TRUE** |
| ***0*** | ***14*** | *1313* | *1320* | *1339* | *True* | *True* | *True* | *3.6* | *28.4* | *31.0* | *True* | *True* | *True* | ***TRUE*** |
| **0** | **17** | 1313 | 1253 | 1339 | False | True | True | 3.6 | 16.7 | 31.0 | True | True | True | **Undetermined** |
| **0** | **20** | 1313 | 1387 | 1339 | True | True | True | 3.6 | 5.1 | 31.0 | True | True | True | **TRUE** |
| **0** | **30** | 1313 | 1279 | 1339 | False | True | True | 3.6 | 3.4 | 31.0 | False | True | True | **FALSE** |

e. sensitivity to N(t0)

|  | Productive performance | | | Comparison | | | Ecological performance | | | Comparison | | | Hypothesis 1 |
| --- | --- | --- | --- | --- | --- | --- | --- | --- | --- | --- | --- | --- | --- |
| N(t_0_) | AO | HO | RO | AO<HO | HO<RO | AO<RO | AO | HO | RO | AO<HO | HO<RO | AO<RO |
| **25** | 1313 | 1321 | 1309 | True | False | False | 3.2 | 26 | 31 | True | True | True | **Undetermined** |
| ***30*** | *1313* | *1321* | *1339* | *True* | *True* | *True* | *3.6* | *28* | *31* | *True* | *True* | *True* | ***TRUE*** |
| **35** | 1313 | 1321 | 1369 | True | True | True | 4.1 | 30 | 31 | True | True | True | **TRUE** |

**Table S5.2** Sizes of the viability tubes *Θ(VT)* for different settings of parameter values and for the three action-oriented (AO), habitat-oriented (HO) and result-oriented (RO) scenarios. For each set of parameters, hypothesis 1 was either qualified as True, False or Undetermined. results corresponding to the set of parameter used in the first step of the study is shown in *italic*

a. Sensitivity to cattle density u# and clutch size fb

| u^#^ | f^b^ | Θ(VT_HO_) | Θ(VT_RO_) | Hypothesis 2 |
| --- | --- | --- | --- | --- |
| **1** | **3.2** | 0 | 6842 | **True** |
| **1.5** | **2.9** | 0 | 6842 | **True** |
| ***2*** | ***2.5*** | *4997* | *6842* | ***True*** |
| **3** | **1.9** | 4997 | 6842 | **True** |
| **4** | **1.5** | 5494 | 6842 | **True** |
| **5** | **1.1** | 5508 | 6842 | **True** |

c. Sensitivity to grass height threshold h^b^

| h^b^ | h^#^ | Θ(VT_HO_) | Θ(VT_RO_) | Hypothesis 2 |
| --- | --- | --- | --- | --- |
| ***0*** | ***14*** | *4997* | *6842* | ***True*** |
| **5** | **14** | 4972 | 6842 | **True** |
| **7** | **14** | 4894 | 6842 | **True** |
| **10** | **14** | 4765 | 6842 | **True** |
| **12** | **14** | 4677 | 6842 | **True** |
| **13** | **14** | 4677 | 6842 | **True** |
| **14** | **14** | 0 | 6842 | **True** |

d. Sensitivity to grass height threshold h#

| h^b^ | h^#^ | Θ(VT_HO_) | Θ(VT_RO_) | Hypothesis 2 |
| --- | --- | --- | --- | --- |
| **0** | **10** | 0 | 6842 | **True** |
| **0** | **12** | 4990 | 6842 | **True** |
| ***0*** | ***14*** | *4997* | *6842* | ***True*** |
| **0** | **17** | 5070 | 6842 | **True** |
| **0** | **20** | 5729 | 6842 | **True** |
| **0** | **30** | 6459 | 6842 | **True** |

e. Sensitivity to initial population size N(t_0_)

| N(t_0_) | Θ(VT_HO_) | Θ(VT_RO_) | Hypothesis 2 |
| --- | --- | --- | --- |
| **25** | 4997 | 6842 | **True** |
| ***30*** | *4997* | *6842* | ***True*** |
| **35** | 4997 | 6842 | **True** |
