# Supplementary material for: Action versus Result-Oriented Schemes in a Grassland Agroecosystem: A Dynamic Modelling Approach
Source: PLoS One. 2012 Apr 5;7(4):e33257. doi: 10.1371/journal.pone.0033257 (PMC3320605; doi:10.1371/journal.pone.0033257)
Supplement: Table S1 — Parameters used in the grazed grass model. (DOC) [file pone.0033257.s006.doc]

**Table S1.** Parameters of the grazed grass sub-model

| **Parameter** | **Value** |
| --- | --- |
| Biomass and grass height relationship | *h* = 8.10^-10^ *B*  r² = 0.98, n=15, p = 2.2 10^-16^ |
| Monthly vectors:  Grass growth (g.m^-²^.month^-1^) *r_G_*  Scenescence rate (month^-1^) *r_S_*  Decay rate (month^-1^) *r_D_* | [0.009 , 0.009, 0.126, 0.137, 0.486, 0.45, 0.45, 0.45, 0.45, 0.45, 0, 0]  [0, 0, 0, 0.93, 1.125, 1.5, 0.75, 0.75, 1.5, 1.5, 0.75, 0.75]  [0, 0, 0, 1.482, 1.105, 1.3, 1.95, 0.65, 0.65, 0.65, 0, 0] |
| Cattle unit feed requirement *q* (g Organic Mater month^−1^ LU^-1^) | 3.8 10^5^ |
| Attenuation coefficient *β*  Specific leaf area *μ* | 0.5  0.02  Both values (*β,* *μ*) from Hutchings and Gordon (Hutchings & Gordon 2001) |

**Bibliography**

Hutchings, N. J. & Gordon, I. J. (2001) *A dynamic model of herbivore-plant interactions on grasslands*. *Ecological Modelling,* **136,** 209-222.
