# Supplementary material for: Action versus Result-Oriented Schemes in a Grassland Agroecosystem: A Dynamic Modelling Approach
Source: PLoS One. 2012 Apr 5;7(4):e33257. doi: 10.1371/journal.pone.0033257 (PMC3320605; doi:10.1371/journal.pone.0033257)
Supplement: Table S2 — Parameters used in the bird model. (DOC) [file pone.0033257.s007.doc]

**Table S2.** Parameters used in the bird model

| Parameters | Value | Reference |
| --- | --- | --- |
| Mean Chick survival, *s_1_* | 0.45 | (Peach, Thomson & Coulson 1994) |
| Adult survival, *s_2_* | 0.70 | (Peach, Thomson & Coulson 1994) |
| Adult clutch size, *f_2_^max^* | 4.20 | (Ottvall 2004) |
| Proportion of breeding females, α | 0.75 | (Ottvall 2004) |
| Sex-ratio *σ* | 0.50 | (Ottvall 2004) |
| Daily nest survival for 1 LU/ha, η | 0.990 | (Beintema & Muskens 1987) |
| Incubation length, *t^inc^* | 26 | (Kooiker 1993) |
| Incubation month | April | (Durant *et al*. 2008) |
| Chick rearing month | May | (Durant *et al.* 2008) |
| Minimal viable grass height *h^b^* (cm) | 0 | (Durant *et al.* 2008) |
| Maximal viable grass height *h^#^* (cm) | 14 | (Durant *et al.* 2008) |

**Bibliography**

Beintema, A. J., G.J.D.M, Muskens (1987) *Nesting success of birds breeding in Dutch agricultural grassland*. *Journal of Applied Ecology,* **24,** 743-758.

Durant, D., Tichit, M., Fritz, H. & Kerneis, E. (2008) *Field occupancy by breeding lapwings Vanellus vanellus and redshanks Tringa totanus in agricultural wet grasslands*. *Agriculture Ecosystems & Environment,* **128,** 146-150.

Kooiker, G. (1993) *Phenology and breeding biology of lapwing (Vanellus vanellus) - Results of a 17 year study in NW Germany*. *Journal Fur Ornithologie,* **134,** 43-58.

Ottvall, R. (2004) Population ecology and management of waders breeding on coastal meadows. Ph.D. Thesis, Lund.

Peach, W. J., Thompson, P. S. & Coulson, J. C. (1994) *Annual and Long-Term Variation in the Survival Rates of British Lapwings Vanellus-Vanellus*. *Journal of Animal Ecology,* **63,** 60-70.
